# Green Synthesis of Copper Oxide Nanoparticles from the Leaves of *Aegle marmelos* and Their Antimicrobial Activity and Photocatalytic Activities

**DOI:** 10.3390/molecules28227499

**Published:** 2023-11-09

**Authors:** Syed Ghazanfar Ali, Uzma Haseen, Mohammad Jalal, Rais Ahmad Khan, Ali Alsalme, Hilal Ahmad, Haris Manzoor Khan

**Affiliations:** 1 Department of Microbiology, Jawaharlal Nehru Medical College, Aligarh Muslim University, Aligarh 202002, India; 2 Department of Chemistry, Aligarh Muslim University, Aligarh 202002, India; 3 Department of Chemistry, College of Science, King Saud University, Riyadh 11451, Saudi Arabia; 4 SRM Institute of Science and Technology, Kattankulathur, Chennai 603203, India

**Keywords:** *Aeglemarmelos*, *C. albicans*, copper oxide, nanoparticles, SEM, TEM

## Abstract

The leaves of the Aegle marmelos plant were used for the green synthesis of copper oxide nanoparticles and further characterized by different techniques, including (Ultra Violet-Visible) UV-Vis, Scanning electron microscopy (SEM), Energy dispersive X-ray (EDX), Transmission electron microscopy (TEM) and X-ray diffraction (XRD). The UV-Vis showed a peak at 330 nm, which may be due to the Surface Plasmon Resonance phenomenon. XRD analysis showed the crystalline nature of copper oxide nanoparticles (CuO NPs). In contrast, SEM showed that nanoparticles were not aggregated or clumped, EDX showed the presence of elemental copper., and further, the TEM analysis revealed the average particle size of copper oxide nanoparticles to be 32 nm. The Minimum Inhibitory Concentration (MIC) for *Escherichia coli* (*E. coli*) and *Staphylococcusaureus* (*S. aureus*) was found to be 400 µg/mL, whereas for *Candida albicans* (*C. albicans*) and *Candida dubliniensis (C. dubliniensis)* it was 800 µg/mL. The zone of inhibition in the well diffusion assay showed the antimicrobial activity of copper oxide nanoparticles, and it also showed that as the concentration of copper oxide nanoparticles increased, the zone of inhibition also increased. Further, the electron microscopic view of the interaction between copper oxide nanoparticles and *C. albicans* cells showed that CuO NPs were internalized and attached to the cell membrane, which caused changes in the cellular structure and caused deformities which eventually led to cell death. The prepared CuO NPs showed significant photocatalytic degradation of organic dyes in the presence of sunlight.

## 1. Introduction

Nanotechnology has emerged as a pivotal and transformative force within contemporary scientific endeavors, exerting a profound influence on various domains of modern science [1,2]. One of the areas where its influence is most palpable and dynamic is within the realm of material sciences. The advent of nanotechnology has ushered in a new era of exploration and innovation, fundamentally altering the way researchers approach and manipulate materials at the nanoscale, which ranges from 1 to 100 nm in size [3,4]. It is not only confined to engineering or physics but is actively involved in pharmaceutical, cosmetics, space industries, environmental sciences, and electronics [5]. Due to the presence of nanotechnology in different aspects, it is believed that it can be an innovative invention that can play a pivotal role in improving human health and can be a good source for enhancing human well-being [6]. Various techniques are employed to synthesize nanoparticles, encompassing physical, chemical, and biological methods. Physical processes often necessitate elevated temperatures and pressures, posing energy-intensive challenges, while chemical methods, which were widely utilized, can involve hazardous chemicals, extended reaction times, and the absorption of toxic byproducts onto the nanomaterial surface. These limitations underscore the significance of exploring alternative, greener synthesis approaches, such as biological methods, which offer an eco-friendlier and more sustainable route to nanoparticle production, often harnessing the capabilities of biological entities like bacteria or plants to craft nanoparticles with reduced environmental impact and enhanced biocompatibility [7,8]. The green synthesis of nanoparticles, which harnesses the properties of plants or plant components, has emerged as the preferred method in recent years. This approach offers a simple, environmentally friendly alternative that does not necessitate external pressure or high temperatures, eliminating the need for hazardous chemicals and averting the release of toxic byproducts. By leveraging the natural capabilities of plants, this method not only contributes to eco-friendliness but also enhances biocompatibility, making it an increasingly favored choice for the sustainable and safe production of nanoparticles with a wide range of applications in fields such as medicine, electronics, and materials science [9,10]. The utilization of plants in nanoparticle synthesis, coupled with the absence of pollutants and environmentally harmful substances, has garnered substantial importance in the realm of material sciences. This green synthesis method not only aligns with the growing global emphasis on sustainability and eco-friendliness but also addresses critical challenges associated with traditional synthesis techniques. Its ability to produce nanoparticles with tailored properties and minimal environmental impact has positioned it as a pivotal and promising approach for advancing materials science, offering innovative solutions to various industries while promoting a greener and more responsible scientific endeavor [9,11]. Copper oxide is a simple copper-containing compound that has a variety of characteristics [7]. Copper is a low-cost metal and is much cheaper than gold or silver [12]. Nanoparticles derived from inorganic metals, like copper oxide nanoparticles (CuO-NPs), have gained notable prominence in recent times for several compelling reasons. Their easy accessibility, cost-effectiveness, and pronounced antimicrobial properties have collectively contributed to this heightened interest. Copper oxide nanoparticles have demonstrated the potential to act as potent antimicrobial agents, effectively inhibiting the growth of a wide range of harmful microorganisms, including bacteria and fungi. This multifaceted appeal, combining economic feasibility and antimicrobial efficacy, underscores their growing significance in various applications, such as water treatment, healthcare, and materials science, where the need for efficient and affordable antimicrobial solutions is increasingly pressing [13]. Indeed, copper oxide nanoparticles have garnered a substantial amount of attention and interest due to their versatility and applicability in various fields beyond just antimicrobial properties. Their unique properties make them valuable in a wide range of applications, including solar cells, photocatalysis, supercapacitors, etc. The adaptability and performance of copper oxide nanoparticles in these diverse applications highlight their significance in addressing various technological and environmental challenges. Researchers continue to explore and optimize their properties for enhanced performance in these and other areas, making them an exciting area of study in materials science and nanotechnology [12,13,14]. Copper oxide nanoparticles, with their potent antimicrobial properties, have found valuable applications in the field of healthcare, making significant contributions to various aspects of medical science and nanomedicine. In diagnostic applications, copper oxide nanoparticles can be integrated into biosensors and diagnostic assays to enhance their sensitivity and accuracy. They can be used to detect specific pathogens or biomarkers associated with diseases, aiding in early and precise diagnosis. They can be used as drug carriers to deliver medications directly to specific sites within the body, offering targeted and controlled drug release. Overall, copper oxide nanoparticles play a pivotal role in advancing healthcare by offering innovative solutions for infection prevention, diagnostics, drug delivery, and therapeutics. Their unique combination of antimicrobial properties and nanoscale features positions them as valuable tools in the fight against human pathogens and the improvement of medical practices [15,16,17].

Multi-drug resistance in the microbial world is causing serious health concerns worldwide. The conditions due to antibiotic resistance in microorganisms are so bad that the World Health Organization and Center for Disease Control and Prevention have declared emerging antibiotic resistance as one of the primary global concerns [18]. The United States Center for Disease Control and Prevention (CDC) estimated that two million people in the United States are affected by antibiotic-resistant infections every year and 23,000 die [19]. The infection due to the microorganisms sometimes becomes fatal. MRSA infection which involves the skin can cause necrotizing pneumonia fasciitis which can lead to serious conditions [20,21]. Similarly, *P. aeruginosa,* an opportunistic pathogen, can cause surgical wound infections, respiratory tract infections, nosocomial infections, Urinary tract infections, and secondary infections in HIV-infected patients [22]. *Klebsiella pneumoniae* causes bloodstream, urinary and respiratory tract infections which are more common in hospitals in pre-term infants, impaired immune system patients and elderly patients [23,24]. Different species of Candida cause candidiasis with *C. albicans* being the major causal agent of the disease worldwide [25]. It is the most frequently isolated species from patients [26]. Multi-drug resistance to different antifungals in other species of *Candidaviz*. *C. glabrata* and *C. auris* have been reported [27].

*Aegle marmelos* is a deciduous flowering plant that belongs to the family *Rutacae*. The plant has several medicinal properties, the methanolic extract of the fruits possesses the potential to decrease intestinal propulsion in rats [28]. Chloroform extract of roots of *A. marmelos* showed antibacterial properties which were comparable to the ciprofloxacin [29]. The aqueous and ethanolic extracts both have shown antibacterial activity against *E. coli*, *Pseudomonas aeruginosa*, *Staphyococcusauereus*, and *Bacillus subtilis* [30]. The leaf extract of *A. marmelos* has shown increased activity for the enzymes SOD, catalase, and glutathione peroxidase in normal mice and diabetic rats [31]. Studies have shown the green synthesized copper nanoparticles from leaves of *Aegle marmelos* have shown potent antibacterial activity against *Proteus*, *S. aureus*, *E. coli and Salmonella* [32]. Due to the medicinal importance of the *Aegle marmelos* plant, copper oxide nanoparticles from the leaf extract of *Aegle marmelos* have been synthesized following a well-established procedure, characterized and systematically studied for antimicrobial and dye degradation activity [33,34,35].

## 2. Results

### 2.1. UV-Vis

**Figure 1:**
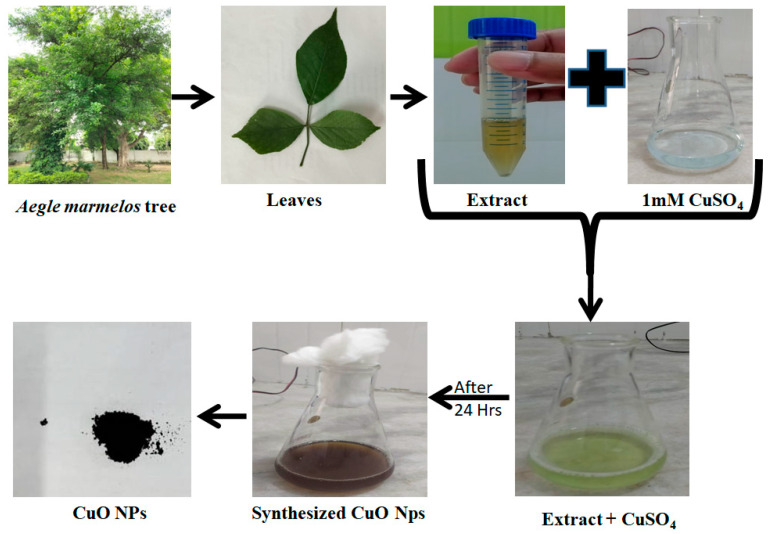
Schematic illustration of synthesis of copper oxide nanoparticles.

**Figure 2:**
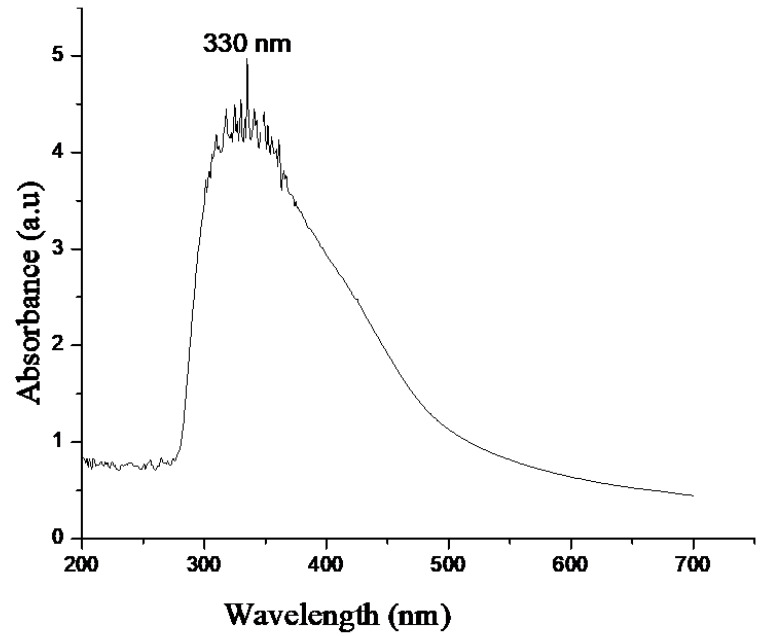
UV-Vis spectra of copper oxide nanoparticles.

In Figure 1, a schematic representation of the formation of copper oxide nanoparticles is depicted. Notably, the change in color serves as the initial indicator of nanoparticle formation. Figure 2 displays the UV-Vis spectrum, showing a peak at 330 nm, which corresponds to the surface plasmon resonance (SPR) of the formed nanoparticles. This SPR peak is a characteristic feature of nanoparticle structures and further confirms the successful synthesis of copper oxide nanoparticles in the study.

### 2.2. SEM with EDX

**Figure 3:**
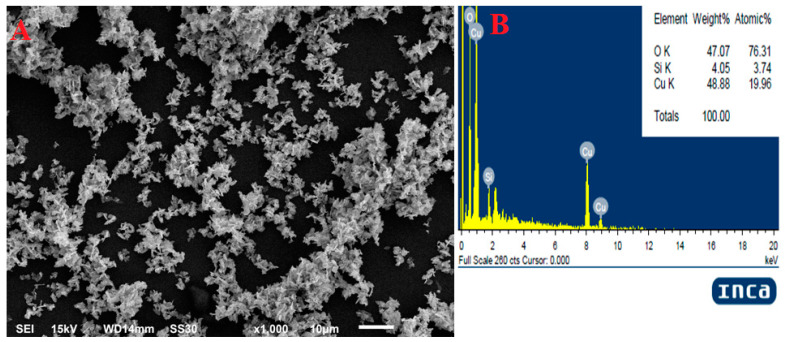
SEM (**A**) and EDX (**B**) of synthesized copper oxide nanoparticles.

In Figure 3A, the Scanning Electron Microscopy (SEM) image provides insight into the surface morphology of the nanoparticles, revealing that they are well-separated and not agglomerated. This even distribution is a critical characteristic of nanoparticles. Figure 3B, on the other hand, displays the Energy Dispersive X-ray Spectroscopy (EDX) data, which indicates the elemental composition of the sample. It clearly confirms the presence of Copper and Oxygen, consistent with copper oxide nanoparticles. Additionally, the detection of traces of Silicon suggests that this element may have originated from the glass coverslip used in the experiment, providing valuable information about potential contaminants or elements introduced during the sample preparation process.

### 2.3. TEM

**Figure 4:**
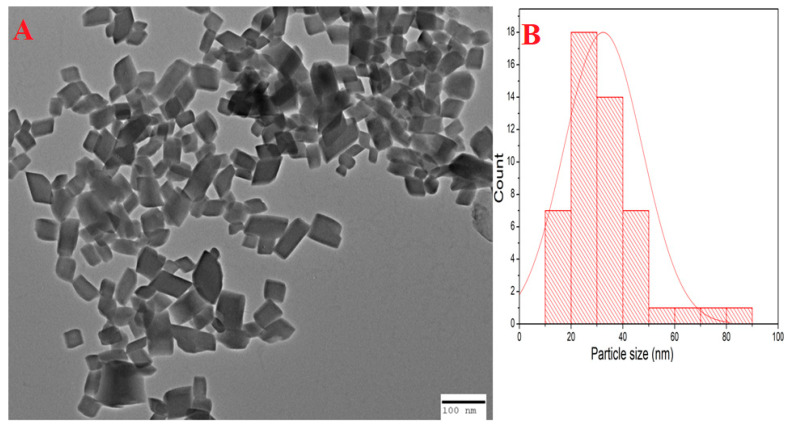
TEM (**A**) and particle size analysis using histogram (**B**) of green synthesized copper oxide nanoparticles.

In Figure 4A, Transmission Electron Microscopy (TEM) is employed to depict the size and shape of the green-synthesized copper oxide nanoparticles. The image shows various shapes of nanoparticles, highlighting the diversity in their morphology. Figure 4B provides additional quantitative information, indicating that the average particle size of these nanoparticles, as measured from the histogram, is approximately 32 nm. This precise size determination is crucial in understanding the characteristics of the nanoparticles and their suitability for specific applications, as it helps to establish their size distribution and uniformity.

### 2.4. XRD

**Figure 5:**
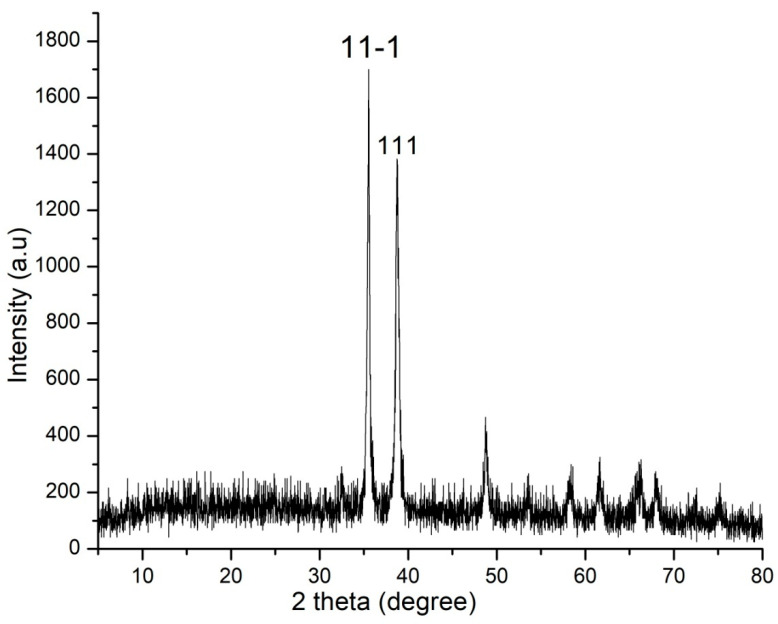
XRD of copper oxide nanoparticles.

Figure 5, which represents X-ray Diffraction (XRD) data, is instrumental in determining the crystalline nature of the copper oxide nanoparticles. The presence of distinct peaks in the XRD pattern is indicative of the crystallinity of the material. In this case, the major peaks observed at 36.2 and 38.4 degrees correspond to the crystal planes 11-1 and 111 of CuO, respectively. This observation suggests that the copper oxide nanoparticles possess a monoclinic crystal structure consistent with the JCPDS-48-1548 reference. The XRD analysis confirms the crystalline nature of the synthesized nanoparticles and provides valuable information about their structural characteristics.

### 2.5. MIC

The Minimum Inhibitory Concentration (MIC) is a crucial parameter in antimicrobial studies, indicating the lowest concentration of a substance (in this case, CuNPs or copper nanoparticles) required to inhibit the growth of specific microorganisms [36,37]. In this study; the MIC of CuNPs against E. coli and S. aureus was determined to be 400 µg/mL. This means that a concentration of 400 µg/mL or higher of CuNPs is required to effectively inhibit the growth of these bacterial strains. Similarly, for *C. albicans* and *C. dubliniensis*, the MIC value was found to be 800 µg/mL. This higher concentration indicates that these fungal strains are less susceptible to the inhibitory effects of CuNPs compared to the bacteria tested. The MIC values are critical in assessing the antimicrobial efficacy of CuNPs against these specific microorganisms and provide valuable information for potential applications in controlling or treating bacterial and fungal infections.

### 2.6. Well Diffusion Assay

**Figure 6:**
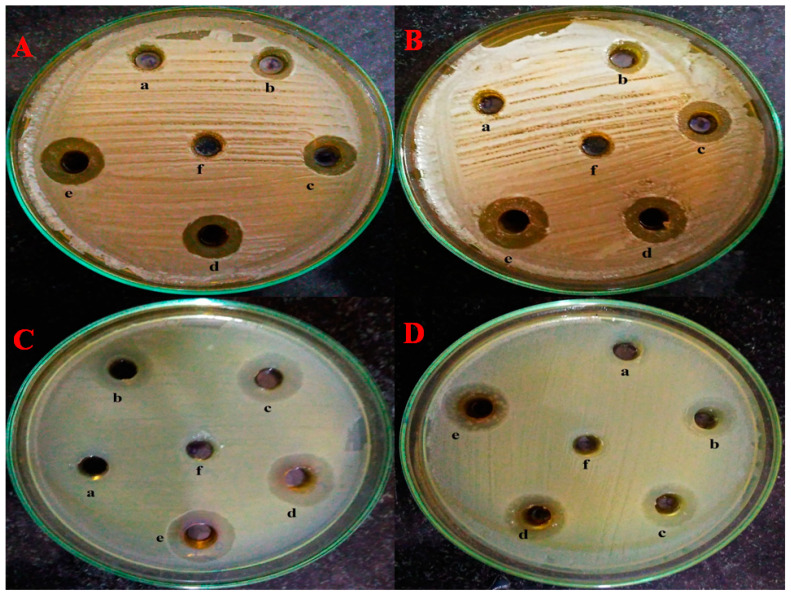
Well diffusion assay. (**A**) = *C. albicans*, (**B**) = *C. dubliniensis*, (**C**) = *E. coli*, (**D**) = *S. aureus* (a) = 62.5 µg/mL, (b) = 125 µg/mL, (c) = 250 µg/mL, (d) = 500 µg/mL, (e) = 1000 µg/mL and (f) = control.

The antimicrobial effect of Copper Oxide nanoparticles (CuO NPs) was evaluated using a well diffusion assay across a concentration range of 62.5 µg/mL to 1000 µg/mL [38]. Figure 6 clearly illustrates that as the concentration of CuO NPs increases, there is a corresponding increase in the size of the inhibition zone. The lowest concentration of 62.5 µg/mL did not show any zone size for the microorganisms tested whereas on increasing the concentration of CuO NPs there is a subsequent increase in zone size and at 1000 µg/mL of CuO NPs the maximum increase in zone size was observed, *E. coli* showed a 22 mm zone size whereas *S. aureus* showed 21 mm of zone size similar to *C. albicans,* which showed a 20 mm zone size whereas *C. dubliniensis* showed a 19 mm zone size. (Supplementary Table S1). This finding indicates a dose-dependent relationship, where higher concentrations of CuO NPs result in a more pronounced antimicrobial effect. Such data are valuable for understanding the effectiveness of CuO NPs in inhibiting the growth of microorganisms and can be instrumental in optimizing their use for various antimicrobial applications.

### 2.7. Interaction of C. albicans Cells with Nanoparticles

**Figure 7:**
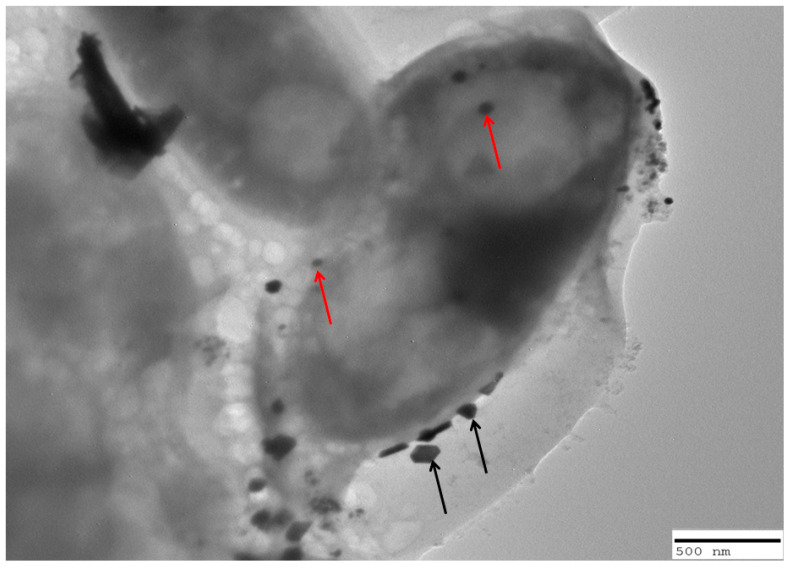
TEM analysis representing interaction of copper oxide nanoparticles with *C. albicans* cells. Red arrows are indicative of internalization of nanoparticles whereas black arrow represents attachment of nanoparticles on the cell surface.

The Transmission Electron Microscopy (TEM) analysis, as depicted in Figure 7, offers insight into the interaction between CuO nanoparticles and *C. albicans* cells. The TEM images clearly illustrate that CuO nanoparticles exhibit a dynamic interaction with the fungal cells. The red arrows in the images indicate instances where CuO nanoparticles are attached to the surface of the *C. albicans* cells, while the black arrows highlight cases where these nanoparticles have been internalized by the cells. This finding suggests that CuO NPs have the potential to interact with and affect the fungal cells both externally and internally, which can be of significance in understanding their antimicrobial mechanisms against *C. albicans* [39].

### 2.8. Photocatalytic Degradation of MB and MO

**Figure 8:**
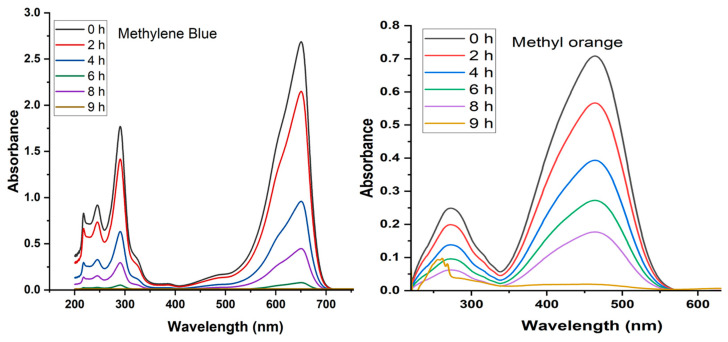
Illustrates the Uv-Vis spectra of dye degradation by CuO NPs.

These analyses provided crucial insights into the effectiveness of CuO NPs as photocatalysts for the degradation of these organic dyes when exposed to sunlight. The dyes are equilibrated with CuO NPs in the absence and in the presence of sunlight for varying time intervals. Figure 8 presents the outcomes of the dye degradation process facilitated by CuO nanoparticles.

## 3. Discussion

The schematic illustration in Figure 1, represents the stepwise synthesis of copper oxide nanoparticles from the leaves of *Aegle marmelos* using copper sulfate. The presence of secondary metabolites in the plant extract was responsible for the formation of copper oxide nanoparticles. Previous studies have suggested the presence of different components such as Alkaloids, carbohydrates, flavonoids, phenols, saponins, steroids and tannins in the leaf extract of *Aegle marmelos* [40].

Initial assessment regarding the formation of copper oxide nanoparticles was conducted using UV-Vis Spectrophotometry, the change in colour was the first indication of formation of nanoparticles. The sharp peak at 330 nm during UV-Vis was due to the surface Plasmon resonance phenomenon (Figure 2). Our results of characterization related to UV-Vis are in accordance with the earlier studies of Shanmugapriya et al. [41] who showed that UV-Vis of green synthesized copper oxide nanoparticles from *Withaniasomnifera* was 330 nm. Scanning electron microscopy showed that nanoparticles were neither aggregated nor clumped (Figure 3A) and the presence of elemental copper was confirmed by EDX (Figure 3B). Further, the TEM analysis confirmed the average particle size was 32 nm and showed that nanoparticles were of different shapes but most of them were rectangular (Figure 4). X-ray diffraction showed that nanoparticles were crystalline (Figure 5). The results for the XRD are similar to the previous studies of Peng et al., 2020 [42].

The MIC of copper oxide nanoparticles against bacteria and fungus showed that fungus possesses a higher MIC than bacteria. The higher MIC in the case of fungi could be due to the fact that fungi are eukaryotic in origin, own better cell organization, complex in structure, and have superior detoxification systems whereas, on the other hand, bacteria are prokaryotic in origin with a simple cellular structure. The complex structure of fungi allows them to better fight against the antimicrobial [43]. Our results for the MIC are in accordance with the previous studies [44] which have shown the MIC of CuO NPs against fungus was higher than bacteria.

A well diffusion assay showed the antimicrobial activity of green synthesized copper oxide nanoparticles. The zone of inhibition proved that copper oxide nanoparticles possess excellent antibacterial and antifungal activity. Bacterial cells showed antibacterial activity at lower concentrations, whereas fungal cells required higher concentrations of nanoparticles. Bacterial cell membranes possess a net negative charge on the surface, which attracts the positive charge materials [45]. Due to the negative charge on the surface, the electrostatic attraction allows the easy adsorption and penetration of CuO NPs inside the cells. Our results are also in agreement with the previous studies which have shown that at low concentration of copper nanoparticles *C. albicans* cells have no significant effects, whereas at higher concentrations, damage to the cell and changes in cellular morphology was observed [46]

Further, the transmission electron microscopic analysis of the interaction of *C. albicans* cells with CuO NPs showed that copper oxide nanoparticles become attached to the cell surface and some nanoparticles become internalized (Figure 7). We are of the opinion that the internalized nanoparticles would have disturbed the normal machinery of the cell due to which the lysis occurred and secondly, the surface attached cells may probably have created the modification in membrane functions due to which the cell death occurred. It has already been shown that nanoparticles disrupt the cell membrane which affects cell permeability respiration; alter proteins containing sulfur and phosphorous which eventually affects the DNA and finally prevent the development of the immune mechanism [47]. Studies have shown that the nanoparticles that pass the cell wall barrier of *C. albicans* directly interact with the integral component ergosterol which is responsible for the cell membrane permeability, fluidity and integrity [48]. In the case of *C. albicans* the accumulated nanoparticles inside the cells are responsible for the production of reactive oxygen species [49] and damage to cellular components such as DNA, protein and lipids [50] and finally cell death [49,51].

In the presence of CuO NPs the photocatalytic process is primarily driven by hydroxy radicals, recognized as the pivotal reactive oxygen species responsible for dye degradation [52]. These nanoparticles are well-established catalysts in the realm of photocatalytic degradation and the reduction of various contaminants [53]. Control experiments conducted in the absence of CuO NPs demonstrated minimal dye degradation, confirming that the dyes did not degrade significantly in dark conditions. Under direct sunlight in the presence of the catalyst, both MB and MO dyes experienced nearly complete degradation, with degradation percentages of 96% and 99%, respectively, within 9 h (Figure 8). Notably, MO dye degradation slightly surpassed that of MB dyes. Overall, the application of CuO nanoparticles for photocatalysis in the decontamination of MB and MO dyes represents an environmentally sustainable and highly efficient technique. These findings contribute to our understanding of the potential of CuO NPs as effective photocatalysts for the degradation of organic dyes under sunlight irradiation.

## 4. Materials and Methods

### 4.1. Chemicals and Microorganisms

Copper sulfate, methylene blue and methyl orange dyes were purchased from Sigma (Steinem, Germany). Microorganisms (*Candida albicans, Candida tropicalis*, *Escherichia coli* and *Staphylococcus aureus*) were obtained from the Department of Microbiology J. N. Medical College.

### 4.2. Preparation of Aegle Marmelos Leaf Extract

Leaves of *Aegle marmelos* were collected, washed with distilled water and shade dried for a few days. The dried leaves were crushed into pieces, and the pieces were again crushed to fine powder. The powder was further allowed to pass through the muslin cloth so that the large particles could be eliminated. Further, the 10 g of fine powder was mixed with 100 mL of double distilled water and kept in a shaker (rotary) for 10–15 mts at 60 rpm. The aqueous extract was filtered using Whatman no 1 filter paper and the extract was stored at 4 °C for further use [33].

### 4.3. Synthesis of Aegle Marmelos Copper Oxide Nanoparticles (AM-CuO NP’s)

The experiment involved mixing 10 mL of Aegle marmelos leaf extract with 90 mL of a 1 mM Copper (II) sulfate solution. This mixture was left to stir for 24 h, during which a change in color was observed [34]. Following the 24-hour period, the solution was centrifuged at 10,000–12,000 rpm, resulting in the formation of a pellet. This pellet was subsequently dried and subjected to further analysis. This experiment likely aimed to investigate the potential interactions or reactions (oxidation) between the leaf extract and the copper sulfate solution. The obtained particles (yield 82%) were then fully characterized and studied for their properties and possible applications in antimicrobial activities and in the catalytic degradation of dyes taken as model pollutants. Figure 8 illustrates the steps involved during synthesis.

### 4.4. UV–Vis Spectroscopy

The green synthesized copper oxide nanoparticles and the concentration of dyes were analyzed by using a UV-Vis Spectrophotometer (Perkin–Elmer Lambda 25 spectrophotometer, Waltham, MA, USA) operated at a wavelength of 200–700 nm.

### 4.5. SEM with EDX

SEM (JSM-6510LV, Kyoto, Japan) equipped with EDAX (INCAx-actSN: 56756) was performed in order to know the morphology and elemental composition. Briefly, the nanoparticles were placed on a glass coverslip which was mounted on an aluminum stub, the samples were then analyzed at an accelerating voltage of 15 KV [35]

### 4.6. TEM

TEM (JEOL-2100, Tokyo, Japan) was used to know the size of nanoparticles. Briefly, a drop of suspension was placed on a copper grid and allowed to dry. After drying, the sample was illuminated with electronic radiation under vacuum. The electron beam allowed the detection of a sample [35].

### 4.7. XRD

The crystalline or amorphous nature of copper oxide nanoparticles was confirmed by XRD (Bruker D8 Diffractometer, GmbH, Karlsruhe, Germany) using CuKa radiation (λ = 1.54056Å) in the range of 10° ≤ 2θ ≤ 80° at 40 keV. The peaks obtained were analyzed.

### 4.8. Determination of Minimum Inhibitory Concentration

MIC of Copper oxide nanoparticles was determined using the broth dilution method [36,37]. Briefly, the bacterial/fungal cultures were freshly grown on nutrient agar (NA)/Sabouraud dextrose agar (SDA). Freshly, grown colonies were picked and re-inoculated in NB/SD broth for 12–18 h at 37/28 °C, respectively. The bacterial/fungal suspension was allowed to inoculate the serially diluted copper oxide nanoparticles.

### 4.9. Well Diffusion Assay

The antimicrobial activity of copper oxide nanoparticles was analyzed using a well diffusion assay as previously described [38]. Briefly, Mueller Hinton Agar and Sabouraud Dextrose Agar plates were prepared for bacteria and fungi, respectively. The wells were punched, and different concentrations of copper oxide nanoparticles were added to each well and incubated.

### 4.10. TEM Analysis of Interaction of Copper Oxide Nanoparticles with Candidal Cells

Candidal cell interactions with copper oxide nanoparticles were analyzed using TEM [39]. Briefly, the Sabouraud dextrose broth containing candidal cells (10^6^ cells per mL) was treated with 1000 µg/mL of copper oxide nanoparticles and incubated at 28 °C overnight. After the incubation, Phosphate Buffer Saline (PBS) was used to wash the cells and fixed with 2.5% glutaraldehyde for 24 h. PBS was again used to wash the cells three times after incubation and dehydrated with alcohol. Finally, the cells were placed in PBS and viewed under TEM by placing a drop of cell suspension on a copper grid.

### 4.11. Photocatalytic Activity of CuO NPs

We investigated the photocatalytic capabilities of CuO NPs synthesized through a green method for the degradation of Methylene Blue (MB) and Methyl Orange (MO) dyes under sunlight irradiation. Experimental procedures involved the preparation of 10 mL dye solutions, each with an initial concentration of 10 ppm. CuO NPs were introduced into these solutions at an approximate concentration of 10 mg L^−1^. Subsequently, the reaction mixtures were stirred in darkness for 15 min to ensure the uniform dispersion of CuO NPs. Following this initial step, the reaction mixtures were exposed to natural sunlight in an outdoor setting for various durations: 2, 4, 6, 8, and 9 h. Continuous stirring was maintained throughout to facilitate the photocatalytic process. After the completion of the photocatalytic reaction, CuO NPs were separated from the solution via centrifugation, and the concentrations of MB and MO dyes were quantified using UV-visible spectroscopy. Specifically, the amplitude of absorbance peaks at 650 nm for MB and 465 nm for MO was measured, and variations in dye concentrations were calculated based on the light absorption intensities in the spectra.

## 5. Conclusions

The results of our study showed that the green synthesized copper oxide nanoparticles can be effectively used for antibacterial, antifungal and photocatalyst dye degradation. The *Aegle marmelos*-mediated copper oxide nanoparticles were stable with an average particle size of 32 nm. We are also of the opinion that the toxicological effect of copper oxide nanoparticles should also be analyzed so that the copper oxide nanoparticles can be effectively used on human cells.

## Data Availability

Data are contained within the article.

## Supplementary Materials

The following supporting information can be downloaded at: https://www.mdpi.com/article/10.3390/molecules28227499/s1, Table S1. Representing the zone of inhibition in mm at different concentration of CuO NPs against different microorganism.

## Sample Availability

Sample of the compounds are not available from the authors.
