# Supplementary material for: Green Synthesis of Copper Oxide Nanoparticles from the Leaves of Aegle marmelos and Their Antimicrobial Activity and Photocatalytic Activities
Source: Molecules. 2023 Nov 9;28(22):7499. doi: 10.3390/molecules28227499 (PMC10673068; doi:10.3390/molecules28227499)
Supplement: Supplementary file 1 [file molecules-28-07499-s001.zip › molecules-2632160-supplementary.pdf]

Green Synthesis of Copper Oxide Nanoparticles from the Leaves of *Aegle marmelos* and Their Antimicrobial Activity and Photocatalytic Activities

Syed Ghazanfar Ali [1], Uzma Haseen [2], Mohammad Jalal [1], Rais Ahmad Khan [3], Ali Alsalme [3], Hilal Ahmad [4,*] and Haris Manzoor Khan [1]

[1] Department of Microbiology, Jawaharlal Nehru Medical College, Aligarh Muslim University, Aligarh 202002, India
[2] Department of Chemistry, Aligarh Muslim University, Aligarh 202002, India
[3] Department of Chemistry, College of Science, King Saud University, Riyadh 11451, Saudi Arabia
[4] SRM Institute of Science and Technology, Kattankulathur, Chennai 603203, India
* Correspondence: hilalahmad418@gmail.com

Supplementary Table S1: Representing the zone of inhibition in mm at different concentration of CuO NPs against different microorganism

	**Zone of inhibition (mm) at different concentration of CuO NPs**				
**Microorganisms**	62.5 µg/ml	125 µg/ml	250 µg/ml	500 µg/ml	1000 µg/ml
E.coli	ND	10	14	18	22
S. aureus	ND	10	13	16	21
C albicans	ND	ND	12	15	20
C. dubliniensis	ND	ND	12	14	19

ND= Not detected
